# Correction: Hypothermia Complications and Associated Electrocardiogram (EKG) Findings

**DOI:** 10.7759/cureus.c471

**Published:** 2026-08-14

**Authors:** Shawn Cullin, Simant Shah

**Affiliations:** 1 Department of Emergency Medicine, Inspira Medical Center Mullica Hill, Mullica Hill, USA

This article has been corrected to clarify that the presence of Osborn waves was not confirmed, and is instead a possibility. Multiple numerical and unit errors have also been corrected, along with inconsistent temperature reporting.

